# Improving Quality and Satisfaction With Handover at the Riverside Centre, Hillingdon

**DOI:** 10.1192/bjo.2024.415

**Published:** 2024-08-01

**Authors:** Gabriella Bernstein, Avani Puri, Mellisha Padayatchi

**Affiliations:** Central and Northwest London NHS Foundation Trust, London, United Kingdom

## Abstract

**Aims:**

This project was launched in January 2022 to improve handover between on-call teams and wards, following GMC concerns in 2020 with the out-of-hours handover process. In 2021, a ‘Hospital At Night’ Microsoft Teams evening meeting was successfully introduced. However there remained low satisfaction with other areas of the handover process, including use of paper forms to handover between shifts. The aims of the project were to review the current handover process and improve quality and satisfaction of handover. The target was to improve baseline satisfaction with the handover process by 20% (6 months after change implementation).

**Methods:**

A driver diagram was built to identify factors contributing to quality and satisfaction with handover and develop change ideas.

Qualitative surveys using Likert rating scales were sent to all doctors to explore satisfaction with handover format and quality of information received. Opinions of doctors and the wider MDT were used to develop ideas and evaluate support for change. Surveys were repeated following each cycle.

From July 2022, interventions were introduced and monitored over four QIP cycles. This included an electronic handover in the form of a twice-daily email handover list, which was updated following feedback. Microsoft Teams morning weekend meetings were then introduced and modelled on the existing ‘Hospital At Night' protocol.

**Results:**

Following interventions, the percentage satisfaction with handover format improved from a baseline of 14% and was maintained at an average of 81% across 15 months.

The satisfaction with the quality of handover improved from 36% and was maintained at an average of 97%.

The weekend virtual handover has also been well received with 71% satisfaction. This maintains the satisfaction levels achieved with the ‘Hospital At Night' virtual handover. The involvement of the MDT has been high with 71% of doctors satisfied that the necessary team members are attending.

**Conclusion:**

Introducing a standardised electronic twice-daily handover has improved satisfaction with and quality of handover. It has also improved communication between on-call teams and wards.

The introduction of additional virtual handover meetings at the weekend has also been well received. It allows another opportunity to strengthen clinical leadership and the MDT to work more effectively out-of-hours. Future intervention will be targeted at standardising the content of these meetings and attendance in line with the ‘Hospital At Night' protocol.

We aim to monitor local benefit from these changes, and expand this project to other hospital sites which are not yet using an electronic handover system.

